# Beautification of images by generative adversarial networks

**DOI:** 10.1167/jov.23.10.14

**Published:** 2023-09-21

**Authors:** Amar Music, Anne-Sofie Maerten, Johan Wagemans

**Affiliations:** 1 Department of Brain and Cognition, KU Leuven, Leuven, Belgium; 2 Department of Brain and Cognition, KU Leuven, Leuven, Belgium; 3 Department of Brain and Cognition, KU Leuven, Leuven, Belgium

**Keywords:** computational aesthetics, image preference, image beautification, generative adversarial networks, low-level image features, mid-level image features, aesthetic experience questionnaire

## Abstract

Finding the properties underlying beauty has always been a prominent yet difficult problem. However, new technological developments have often aided scientific progress by expanding the scientists’ toolkit. Currently in the spotlight of cognitive neuroscience and vision science are deep neural networks. In this study, we have used a generative adversarial network (GAN) to generate images of increasing aesthetic value. We validated that this network indeed was able to increase the aesthetic value of an image by letting participants decide which of two presented images they considered more beautiful. As our validation was successful, we were justified to use the generated images to extract low- and mid-level features contributing to their aesthetic value. We compared the brightness, contrast, sharpness, saturation, symmetry, colorfulness, and visual complexity levels of “low-aesthetic” images to those of “high-aesthetic” images. We found that all of these features increased for the beautiful images, implying that they may play an important role underlying the aesthetic value of an image. With this study, we have provided further evidence for the potential value GANs may have for research concerning beauty.

## Introduction

For millennia, philosophers have been arguing about the nature of beauty. Yet after all these years, we still cannot confidently claim to understand what beauty even *is*. Furthermore, the fundamental question of whether beauty is subjective or objective remains unsettled (Sartwell, 2022). In this article, we will use state-of-the-art machine learning techniques to try and create a so-called visual definition of beauty.

### Studying beauty

Throughout history, there have been different conceptions of beauty. From early conceptions focusing on objective properties to later ideas viewing it through a hedonistic and instrumental perspective, there has never been a clear consensus on what beauty really is. This lack of consensus did not prevent early psychologists from attempting to investigate beauty in what they considered an “objective” way. More specifically, the methods developed in psychophysics such as the method of constant stimuli (Fechner, 1860) allowed for scientific investigation of these latent properties. This classic psychophysical approach is purely stimulus driven or, as described by Fechner (1876), *aesthetics from below*. Expanding on these initial frameworks, a school of thought now known as Gestalt psychology appeared. The Gestalt psychologists were specifically interested in how interactions between elements or parts of visual stimuli can evoke a response greater than the sum of their parts (Wagemans et al., 2012). Later psychological research focusing on art and aesthetic appreciation has found that certain principles tend to evoke a certain aesthetic quality across cultures, such as symmetry (Bode, Helmy, & Bertamini, 2017), order and complexity (Van Geert & Wagemans, 2021), and figure-ground contrast (Reber, 2012). Modern research also attempts to incorporate subjective elements of perception, leading to an interactionist paradigm. Building on this, Redies (2015) proposes two modes for the processing of aesthetic experience. The first mode concerns bottom-up perceptual processing based on intrinsic qualities of a visual stimulus. Only if the outcome of the first mode determines the stimulus to be beautiful, the second mode activates. This second mode is a partially top-down process that incorporates individual variables such as cultural background. One influential framework that incorporates the interactionist interpretation of beauty is that of processing fluency formulated by Reber, Schwarz, and Winkielman (2004). In this framework, the aesthetic experience is determined by the fluency with which a perceived object is processed. Processing fluency relies on an interaction of objective properties such as the principles from Gestalt psychology with subjective factors such as personal experiences and expectations.

### Machine learning and beauty

One of the biggest technological advances in recent years is in the field of machine learning. This “deep learning revolution” was sparked by the continuous efforts of researchers and engineers, the increasing availability of big data, and the boost in computing power (Sejnowski, 2018). Because the deep neural networks that have recently been developed have some organizational similarities to the human brain, they can potentially be used as a means to study obscure mental representations in humans (Goetschalckx, Andonian, & Wagemans, 2021; Guo et al., 2016).

Computational aesthetics is defined as the research of human aesthetic decision-making through computational methods (Hoenig, 2005; Valenzise, Kang, & Dufaux, 2022). Since the deep learning revolution, this branch of aesthetics research has become primarily data driven. Many efforts have been invested toward predicting aesthetic scores using convolutional neural networks (Kao, Huang, & Maybank, 2016; Kong, Shen, Lin, Mech, & Fowlkes, 2016; Lee & Kim, 2019; Lu, Lin, Jin, Yang, & Wang, 2014; Lu, Lin, Shen, Mech, & Wang, 2015; Ma, Liu, & Chen, 2017; Wang et al., 2017). Conwell, Graham, and Vessel (2021) showed that aesthetic scores can be predicted by an ensemble of deep neural networks trained on more traditional computer vision tasks such as object and scene recognition, suggesting that these neural networks capture objective stimulus properties that influence aesthetic appreciation. More recently, Hentschel, Kobs, and Hotho (2022) have shown that CLIP captures aesthetic value of images through language. CLIP is a transformer-based neural network that is trained to link captions to the corresponding images and vice versa. They show that CLIP outperforms these more traditional convolutional neural networks in predicting aesthetic scores.

**Figure 1.**
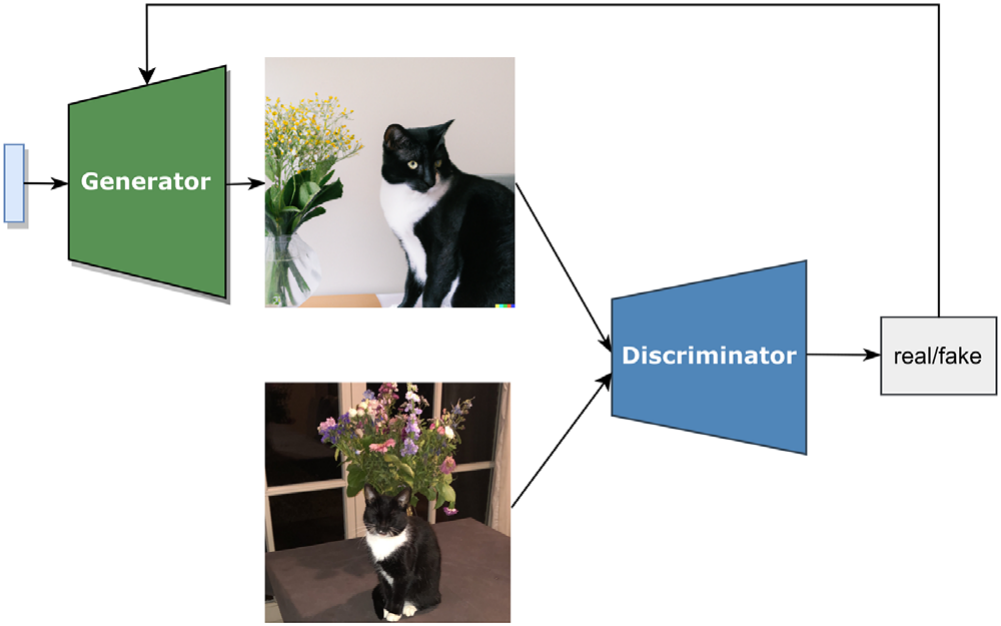
Schematic representation of a typical GAN.

Since the invention of generative adversarial networks (GANs; Goodfellow et al., 2014), generative artificial intelligence has sparked a lot of new research avenues in image processing and vision science. The purpose of a GAN is to learn the probability distribution of complex sensory data, allowing it to generate new sensory samples representative of the original data set (Goetschalckx et al., 2021). In order to learn the probability distribution of a data set consisting of images, a generator network with no access to the image data set is used in combination with a discriminator network that does have access to this data set (Feng et al., 2020). A schematic representation of a traditional GAN can be seen in Figure 1. The generator receives a noise vector as input, which ensures that the generator can produce various samples of the underlying data distribution it models. The generated image is then provided to the discriminator, alongside an existing image. The task of the discriminator is to determine which of the two images was the real image and which was produced by the generator. Initially, the generator will output random pixel values. As training progresses, both the generator and discriminator will learn to optimize their “weights,” or parameters, by receiving feedback on their output. Initially, the generator learns that the generated image was not good enough to fool the discriminator, and the discriminator learns that it correctly identified the real image. For the next iteration, the generator has adjusted its internal weights, resulting in an output that resembles the original data set slightly better. Over time, both networks will improve at their respective tasks until the generator becomes so good at creating images that the discriminator is no longer able to reliably distinguish between real and fake.

**Figure 2.**
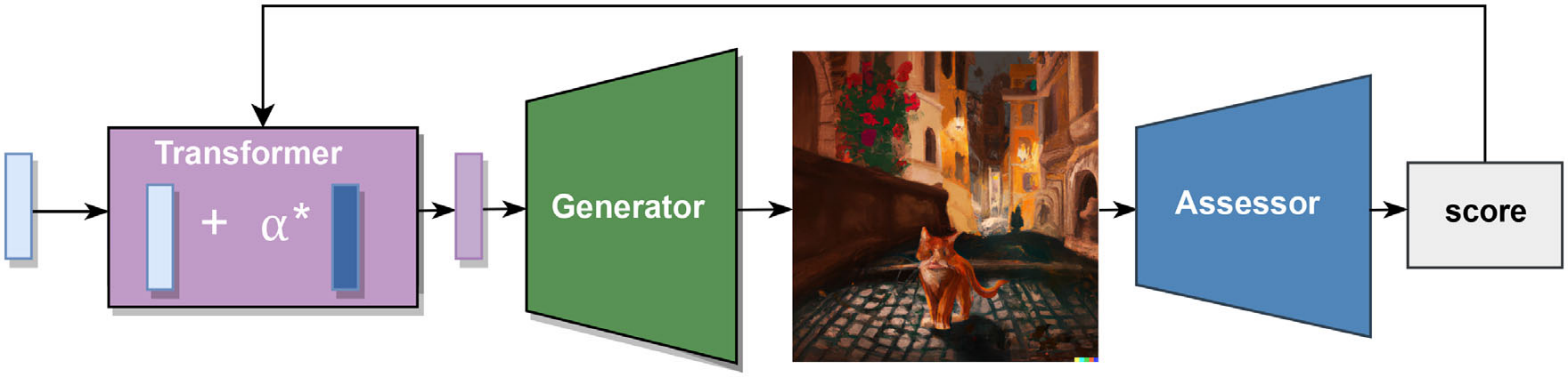
Schematic representation of GANalyze.

Goetschalckx, Andonian, Oliva, and Isola (2019) have developed GANalyze, a framework using GANs that allows us to study cognitive properties by means of so-called visual definitions. In their article, they utilized their model to generate images of varying levels of memorability, aesthetic value, and emotional valence. GANalyze differs from a traditional GAN in several ways: GANalyze includes an “assessor” rather than a discriminator. This assessor is a pretrained convolutional neural network designed for predicting a cognitive property (e.g., image memorability; Khosla, Raju, Torralba, & Oliva, 2015). The images that the generator produces are fed to the assessor, which predicts a memorability score for every image. This score is used as feedback to improve the generated images. This is achieved by including an additional component, the transformer. The transformer is a component of the model that alters the input vector for the generator, in order to steer the output of the generator more toward the desired output (less or more memorable). The transformer receives a random noise vector as input (which would be the input to the generator in a traditional GAN) and combines this noise vector with a second vector that is learned during the training of the GANalyze framework. With feedback from the assessor, this second vector updates its values until it increases the memorability of the generated images. The transformer scales this vector with the α-parameter, which allows the user of GANalyze to either increase or decrease the memorability of generated images. A schematic representation of the GANalyze model can be seen in Figure 2.

Although Goetschalckx et al. (2019) implemented a version of GANalyze for three different assessors (memorability, aesthetic value, and emotional valence), their work focusses on image memorability. They validated GANalyze with the memorability assessor in a behavioral task. The current article validates GANalyze with the aesthetic assessor and tries to uncover a “visual definition” for aesthetics.

### Present study

The goal of this study is to investigate the factors underlying the aesthetic value of images using GANalyze with an aesthetic assessor. We will use GANalyze to generate sequences of images that we hypothesize possess either *more* or *less* aesthetic quality depending on the *α*-value used for their respective generations. By comparing the low-aesthetic images to the high-aesthetic images, we will be able to examine the underlying factors responsible for making an image either more or less beautiful.

However, if we wish to learn something about human aesthetics appraisal from a GAN, we must first validate whether the GAN truly captures what it means for an image to possess aesthetic quality. To do so, we will validate whether human participants tend to agree with GANalyze on which image appears to be more beautiful. We are also interested in whether a person's experience with art and their demographic characteristics affect their proportion of agreement with the GAN. A relation between art experience and agreement with the GAN could have important implications for the validation of the network and the generalizability of the final results.

## Methods

### Stimulus generation

**Figure 3.**
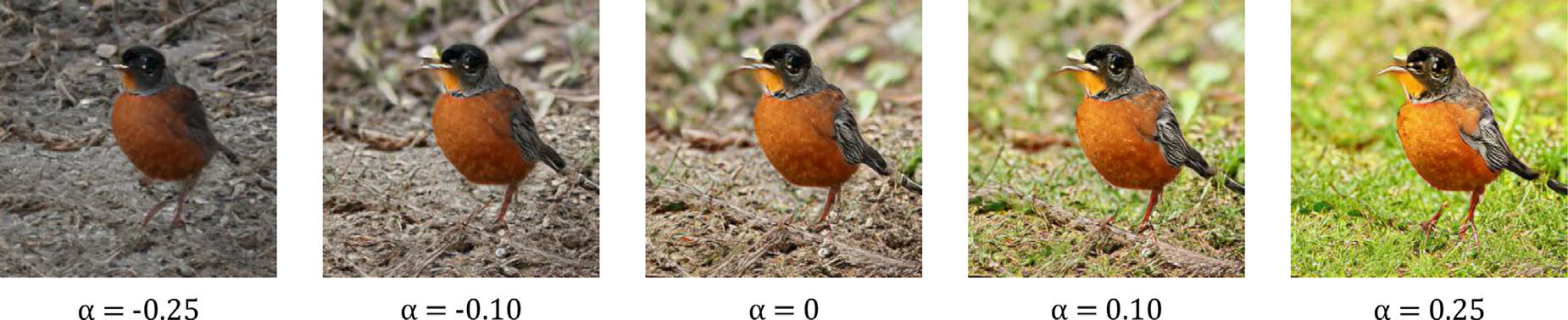
Sample of an image sequence with five *α*-values.

We used GANalyze with AestheticsNet (Kong et al., 2016) as the assessor and BigGAN-256 (Brock, Donahue, & Simonyan, 2019) pretrained on ImageNet (Russakovsky et al., 2015) as the generator to train the GANalyze model for 400,000 iterations. To do this, we used Python 3.6 with the TensorFlow libraries (Abadi et al., 2016). The training resulted in GANalyze being able to produce image sequences based on 1,000 ImageNet categories. For each ImageNet category, we initially generated three different seeds (e.g., three different images of Siamese cats) to test for an effect of idiosyncratic effects of images on top of semantic category. Within each seed, GANalyze produced 21 images with a supposedly increasing amount of aesthetic value (Figure 3). We picked a broad range of *α*-values with increasing density close to zero to obtain stimuli with obvious changes (e.g., *α* = 0.25) but also subtle changes (e.g., *α* = 0.0025). We chose these values because we hypothesized there to be a ceiling effect where there would be no behavioral differences for extremely high *α*-values due to oversaturation. We also hypothesized that for very low *α*-values, the perceptual difference between stimuli would be too small to detect. The specific *α*-values used in the final experiment were chosen based on the results of a pilot study with a wider variety of *α*s. This pilot followed the same general structure as the full experiment, which is explained below. The main difference in the pilot is the use of the unfiltered data set with 1,000 ImageNet categories, 21 *α*-values, and 3 seeds. This pilot showed us that it would be justified to only use one of the three seeds for each category. More details concerning this pilot study are reported in the Results section.

In total, 511 ImageNet categories were manually excluded from the stimulus set because they contained human faces (which are deliberately warped by BigGAN); insects such as spiders, which might make some participants feel uncomfortable; or for redundancy, such as the disproportionately high number of categories containing different dog breeds. In addition, stimuli that we considered unrecognizable were also excluded as we believed these sequences would not be evaluated in the same way. This resulted in 489 categories, each with one seed and 15 *α*-values, equaling a total of 7,335 stimuli. We grouped the ImageNet categories in eight broader groups based on their content. The groups we created are “animal,” “clothing,” “food,” “indoor,” “nature,” “outdoor,” “tool,” and “vehicle.” These groups were only used to test for an effect of semantic content.

### Questionnaire

To measure the aesthetic experience of each participant, we used a shortened version of the Aesthetic Experience Questionnaire (AEQ; Wanzer, Finley, Zarian, & Cortez, 2020). We have chosen this particular questionnaire based on a number of factors. First of all, the theoretical background of an aesthetic flow experience (Csikszentmihalyi & Robinson, 1990) is valid under our assumed interactionist conception of beauty. Second, this questionnaire seems fitting because the AEQ is designed to be generalizable to all visual aesthetic experiences and is not limited to famous pieces in a museum, as is the case with many other questionnaires. Finally, we picked the AEQ because the authors suggest that it should be possible to reduce the items on the six scales and still end up with a sufficient measurement of aesthetic experience.

As the experience one has with art was not the primary focus of our study, we only used the highest loading items from each of the six scales, resulting in a total of six items at the start of our study in order to save time (see Appendix A). Each item was presented with a 5-point Likert scale, ranging from 0 (*strongly* *disagree*) to 4 (*strongly* *agree*). To test whether this shortened version of the AEQ was sufficient to explain the latent aesthetic experience, we used confirmatory factor analysis. The results of this validation can be seen in Appendix B.

### Behavioral experiment

To determine whether GANalyze can be used to study what it means for an image to be aesthetic, we conducted a behavioral study to test whether human participants indeed preferred the images that GANalyze generated to be more aesthetic over a corresponding base image. This study has been approved by the Social and Societal Ethics Committee of the KU Leuven.

**Figure 4.**
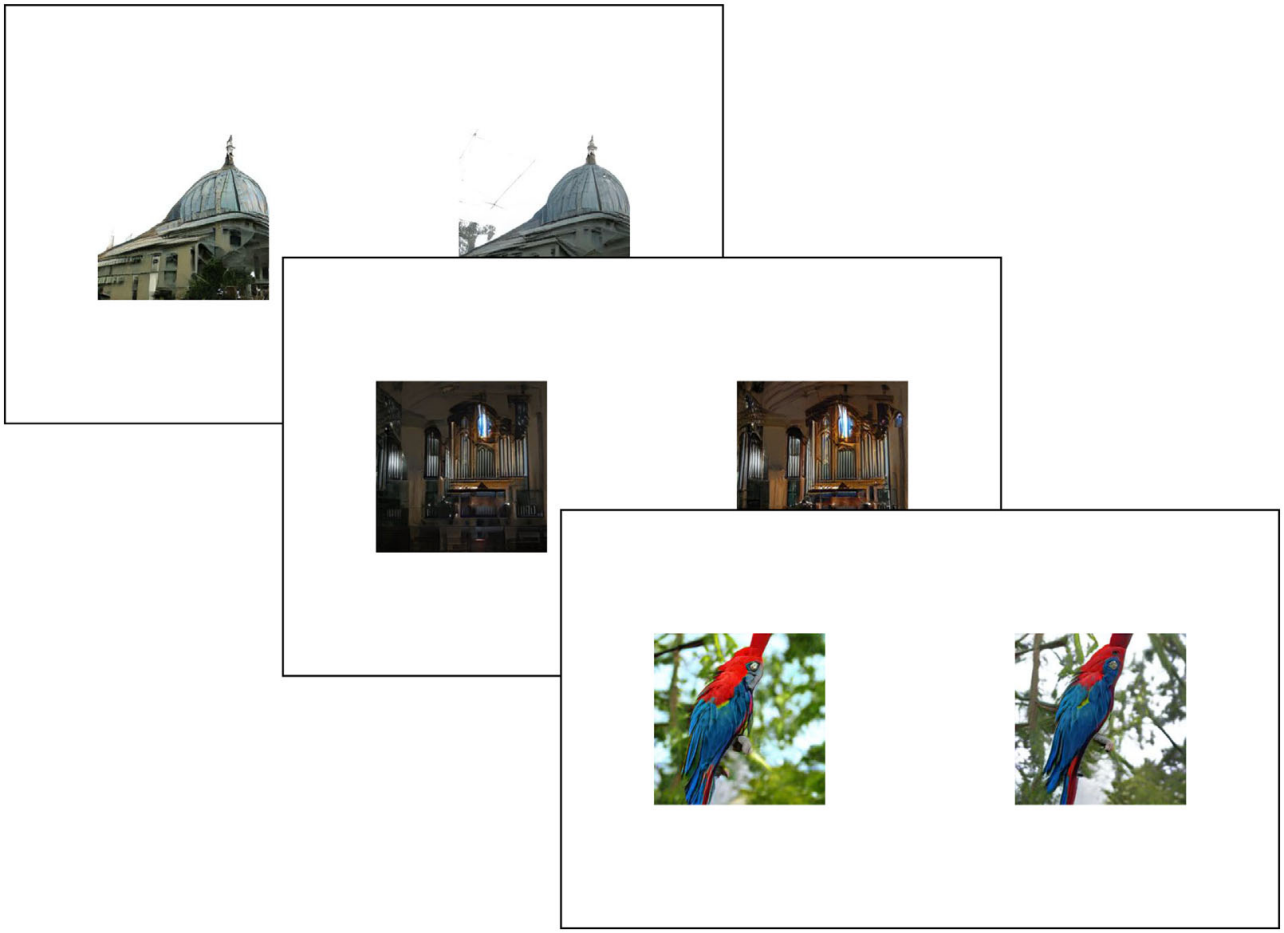
Example of a sequence of trials in the study. Image depicting three typical trials in the experiment. For every trial, one of the two presented images will be the neutral image with α = 0, while the other will have an *α* ranging from −0.25 to 0.25.

The study was hosted on Prolific.co to obtain a large number of data points in a simple online task, ensuring each of the 7,335 stimuli was evaluated around 20 times on average. Individuals younger than 17 or those without normal or corrected-to-normal vision were not allowed to participate. Task instructions were provided on the main Prolific page of this experiment and had to be read before starting the experiment (see Appendix C). The online experiment started with an informed consent document that had to be signed, followed the AEQ. After the AEQ, the main task was initiated. The participants carried out a spatial two-alternatives forced-choice (2AFC) task in which they had to indicate which of the two presented GAN-generated images they considered most aesthetically pleasing. They did this by pressing “F” for the left and “J” for the right image (Figure 4). Participants were instructed that because there was no real correct answer, they should only select the image they personally preferred.

The experiment was created in JavaScript with the jsPsych library (de Leeuw, 2015) for its lightweight implementation in browsers, ensuring we have consistent performance independent of the hardware our diverse participant population may have. Each trial consisted of two images generated from the same ImageNet category. One of the two images was always the base image with α = 0 while the comparison image was, according to GANalyze at least, either more aesthetic (α > 0) or less aesthetic (α < 0). The *α*-value for the comparison image differed for each trial, leading to differences in similarity between the base and comparison images for each trial. An *α*-value close to zero indicates high similarity and, consequently, difficulty to discriminate between the images. An *α*-value much larger or smaller than zero indicates low similarity and therefore easy discrimination. The position of the base and comparison image was randomized for each trial to prevent possible confounding factors. There was no time limit for the trials, and each stimulus pair stayed on the screen until a participant responded. Each 10-minute block was created to contain a roughly uniform distribution of chosen *α*-values and ImageNet categories to ensure that each participant was presented with a comparable stimulus set. Furthermore, participants were never presented with stimuli from an ImageNet category that already appeared once before, meaning that the maximum length for a block was 489 trials: the total number of ImageNet categories used.

As this was a relatively fast-paced and demanding task, we added a short break after 5 minutes to counteract fatigue and loss of concentration. After 10 minutes, the task automatically turned to a screen thanking the participants for their assistance and instructing them to click the link that appeared. Clicking this link redirected them to Prolific, where their successful participation was registered. They were later given a monetary compensation of £1.38 converted to their local currency, only if they had completed the task seriously, thus providing no irrational data. Our criteria for proper task performance are explained further below.

### Data analysis

We analyzed the results from the behavioral task using a variety of methods. First of all, we cleaned the data by removing participants who had a bias for the left/right stimulus > 25% (*n* = 2), performed too close to chance (i.e., < 55% agreement for all *α*-values; *n* = 9), responded too fast to reasonably perceive and judge the stimuli (i.e., median response time (RT) < 500 ms; *n* = 6), and had an exceptionally low number of trials during the 10-minute experiment (i.e., number of trials <50; *n* = 4).

To see whether the different *α*-values indeed represent a gradual change in stimulus difference, we fitted the results of all participants aggregated over *α*-levels with a psychometric function using the quickpsy package (Linares & López-Moliner, 2016). We specified the guess rate and lapse rate of this function as free parameters because we believe aesthetic judgment contains a significant subjective component and therefore considered it unreasonable for participants to agree completely with the network for any *α*-level. Analysis on differences between positive and negative *α*-values was done with polynomial regressions. Finally, we tested for potential confounding factors using a simple two-way analysis of variance (ANOVA).

#### GANalyze parameters

To visualize what makes an image beautiful, we analyzed the image features of the generated images. This is a unique application GANs offer to cognitive science, allowing for substantial control over stimuli that are typically hard to obtain without sacrificing ecological validity (Goetschalckx et al., 2021). Using the Pillow (Clark, 2015) and pyaesthetics (Gabrieli, Bornstein, Setoh, & Esposito, 2023) libraries in Python, we summarized each of the 7,335 images into a single value for various low- and mid-level image features. Based on the study by Ke, Tang, and Jing (2006), we decided to compare the mean brightness, contrast, sharpness, and saturation of all images aggregated over their *α*-values, allowing us to track the evolution of these features as the aesthetic value of their corresponding images increases. In addition, we measured mid-level features such as colorfulness, visual complexity, and symmetry. To measure brightness, we computed the mean pixel values of all images converted to grayscale, resulting in high and low values for bright and dark images, respectively. For contrast, we computed the standard deviation of the pixel values in all grayscale images, resulting in high values for high contrasts and low values for low contrasts. Sharpness was computed by taking the mean value of the grayscale images transformed with a 3 × 3 Laplacian edge-detection filter. We measured the saturation by first converting the images from RGB to HSV and then taking the mean value of only the resulting saturation channel. For the mid-level features, we used functions from pyaesthetics. Colorfulness was evaluated using a metric originally described by Hasler and Suesstrunk (2003) for use in RGB color space. We computed visual complexity with quadratic tree decomposition analysis. This method recursively breaks down an image into smaller subregions based on the variation in pixel values in the created quadrants. Visual complexity is then derived from the resulting total number of quadrants. Finally, we computed symmetry also using quadratic tree decomposition, although here its function was to compare the left and right or top and bottom part of the image. All resulting values were centered and standardized.

To formally investigate the effect of the low- and mid-level features on aesthetic appreciation, we performed a multiple linear regression on aesthetic appreciation using all low- and mid-level features as predictor variables. As a cross-validation measure, we additionally fitted a predictive ridge regression model with an 80–20 train–test split to address potential multicollinearity and to serve as an additional measure of generalizability. Importantly, these models are fitted *across* ImageNet categories in order to be able to generalize these effects (i.e., not being specific to a particular category).

In addition, we investigated how the color distributions change as the aesthetic value of the images increases. We did this by plotting histograms for the individual RGB-values of separate images in an image sequence defined by the ImageNet category. We did this for five random image sequences separately rather than averaging over all images for different *α*-levels. Each image sequence is made up of a unique color composition that may affect its aesthetic value in different ways. Averaging over all images for a given *α*-level would then obscure the valuable information that the evolution of single image sequence could offer.

To investigate higher-level features, we took two approaches. In the first approach, we filtered all image sequences to select only the 95th percentile with the smallest change in low-level features. Increasing aesthetic evaluations for these image sequences after controlling for low-level features would imply an effect of higher-level features. In the second approach, we filtered all image sequences to select only the 95th percentile with the largest difference between the aesthetic ratings of α = 0.25 and α = 0.10. This is done to find the sequences that show a decrease in aesthetic quality despite an increase in the low-level features. This would be evidence for superfluous modifications that end up making the final images appear less beautiful overall. To ensure that this effect is not caused by decreasing realism, we computed mean Fréchet inception distance (FID) for the *α*-values as a measure for realism (Parmar, Zhang, & Zhu, 2022).

As we have not developed a method to formally investigate high-level image features in this study, we simply looked at the resulting image sequences to find out what changes as the aesthetic value increases. We did not draw any specific conclusions from this method due to its subjective and post hoc nature. We did, however, use our findings here to contextualize and nuance the role of the low- and mid-level features we found.

## Results

### Pilot

We conducted a preliminary pilot version of the experiment to find reasonable variables to use for the final experiment. The pilot consisted of 10 participants who completed a version of the experiment with three seeds for each image and 21 *α*-levels. With a generalized linear model, we concluded that there was no effect of seed (*p* = 0.38). With a visual inspection on the effects of *α*-values on agreement, we decided to remove six moderate *α*-values that appeared redundant in showing the relation in order to have better measurements for the useful values.

### Participants

After removing participants who did not satisfy the criteria listed above, our total number of participants was 542 (*M_age_* = 31.17,  *SD_age_* = 11.00,  234 female) from 44 countries across the world.

### Behavioral task

**Figure 5.**
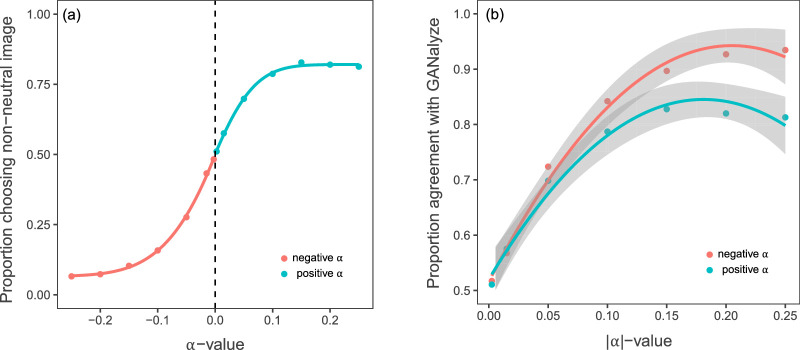
Behavioral results from the image rating task. (a) Psychometric function for the proportion of chosen nonneutral images, illustrating that the estimated threshold value is approximately 0. Choosing the nonneutral image for a negative *α*-trial corresponds with a disagreement with GANalyze and vice versa. Error bars were too small to display in the figure. (b) Proportion of agreement with GANalyze with positive and negative *α*s taken together, fitted with quadratic regression.

To test the effect of different *α*-values on image preference, we fitted a psychometric function on the data (Figure 5a). The figure shows that the relation between the *α*-value used for image generation and the behavioral responses can be fitted quite well with the typical psychometric function commonly found in psychophysical discrimination tasks. As expected, the estimated point of subjective equality for the neutral and nonneutral image is at α = 0 (99% CI [ − 0.00,  0.00]). The estimated guess rate (equivalent to the lapse rate of negative *α*s) is 0.07 (99% CI [0.07,  0.08]) and the estimated lapse rate is 0.17 (99% CI [0.17, 0.18]).

We found a difference between the rating of high-|α| images for positive and negative *α*-values (Figure 5b). People tend to agree more with GANalyze when it generates images that are supposed to be of lower aesthetic value compared to the neutral image. This difference is especially pronounced for the extreme variations. Table 1 shows the results of our polynomial regressions. A quadratic regression with the predictors |α|, |α|^2^ and dummy variable positive explained 96.5% of the observed variance. |α| and |α|^2^ both significantly predicted the proportion agreement with GANalyze. The positive variable was also significant, indicating an asymmetry in the appreciation of the generated high- and low-aesthetic images. We also fitted a cubic regression model, although the marginally better fit could not be justified with the addition of another polynomial to the equation (Δ*R*^2^ = −0.01, *p* = 0.088).

**Table 1.** Polynomial regressions. *Notes*: A significant *b*-weight indicates the semi-partial correlation is also significant. *b* represents unstandardized regression weights. *sr*^2^ represents the semi-partial correlation squared. Square brackets are used to enclose the lower and upper limits of a confidence interval. *p < 0.05, **p < 0.01.

Predictor	*b*	*b* 95% CI	*sr* ^2^	*sr* ^2^ 95% CI	Fit
(Intercept)	0.55**	[0.50, 0.59]			
|α|	3.87**	[3.05, 4.69]	.39	[.07, .72]	
|α|^2^	−10.03**	[−13.28, −6.78]	.17	[−.01, .34]	
positive	−0.05*	[−0.09, −0.02]	.03	[−.02, .09]	
					*R* ^2^ = .965**
					95% CI [.85, .98]
					
(Intercept)	0.53**	[0.48, 0.57]			
|α|	5.28**	[3.46, 7.10]	.12	[−.01, .25]	
|α|^2^	−25.05*	[−43.02, −7.08]	.03	[−.01, .07]	
|α|^3^	39.63	[−7.14, 86.41]	.01	[−.01, .03]	
Positive	−0.05**	[−0.09, −0.02]	.03	[−.01, .08]	
					*R* ^2^ = .975**
					95% CI [.87, .98]

A two-way ANOVA with broad category labels and position of the higher-|α| image as predictors revealed that there was no effect of broad category, *F*(7,  215) = 0.009,  *p* = 1, and no effect of stimulus position, *F*(1,  215) = 0.000,  *p* = 0.99.

### GANalyze parameters

**Figure 6.**
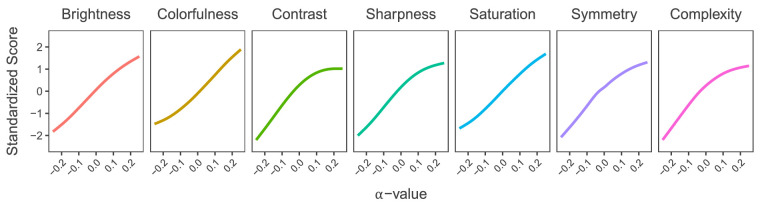
Image features by *α*-value. Figure depicting the change in average brightness, colorfulness, contrast, sharpness, saturation, symmetry, and visual complexity from low-*α* to high-*α* images, illustrating that an increase in these features correlates with aesthetic preference. Values have been transformed from pixel intensities to *z*-scores for comparability.

The results from analyzing the image features of the 7,335 stimuli *within* their respective ImageNet categories are displayed in Figure 6. Brightness, colorfulness, contrast, sharpness, saturation, symmetry, and visual complexity all increase along with the *α*-values. Notably, the curves for contrast, sharpness, symmetry, and complexity start slowing down at high positive *α*-values. This asymmetrical shape was not expected and has theoretical implications. A multiple linear regression model using the image features as predictors for *α*-value was able to account for 62% of observed variance.

**Figure 7.**
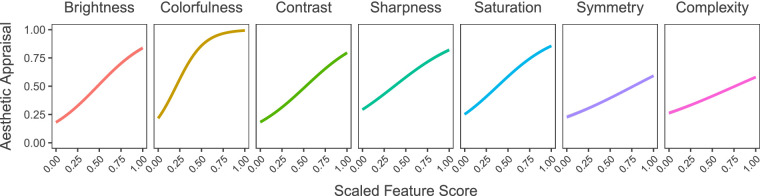
Effects of image features on aesthetic appraisal. Figure depicting the effect of each feature on aesthetic appraisal. The raw scores for the features have been scaled from 0 to 1 for comparability. All features appear to increase aesthetic appraisal.

Figure 7 shows the effects of the image features on the aesthetic appraisal *across* ImageNet categories. The results of multiple linear regression on these features can be seen in Table 2. These results show that all of our computed features apart from symmetry significantly contribute to the aesthetic appreciation of images in our data set.

**Table 2.** Multiple linear regression on image features across ImageNet categories. *Notes*: Residual standard error: 0.2109 on 6,838 degrees of freedom. Multiple *R*^2^: 0.5176. Adjusted *R*^2^: 0.5171. *F*-statistic: 1,048 on 7 and 6,838 *df*, *p* < 2.2e-16.

Predictor	*b*	*SE*	*t*	*p*
(Intercept)	−0.24	0.01	−17.977	0.000
Brightness	0.58	0.02	29.126	0.000
Contrast	0.37	0.02	22.030	0.000
Sharpness	0.49	0.02	21.355	0.000
Saturation	0.39	0.02	15.742	0.000
Colorfulness	0.58	0.04	14.536	0.000
Symmetry	−0.01	0.02	−0.289	0.772
Visual complexity	−0.19	0.02	−9.078	0.000

The *R*^2^ value of 0.52 shows that these features are indeed involved in making the images either more or less beautiful. Fitting a ridge regression model resulted in an optimal estimated λ-value of 0.001, indicating that the ridge model is nearly equivalent to the multiple linear regression model. Furthermore, using a ridge regression model trained on 80% of the data set to predict the remaining 20% resulted in an *R*^2^ value of 0.52 on the test set, which is equivalent to that of the linear model. The root mean square error of the predictive model is equal to 0.21 on both the training set and the test set, showing that there are no signs of overfitting.

**Figure 8.**
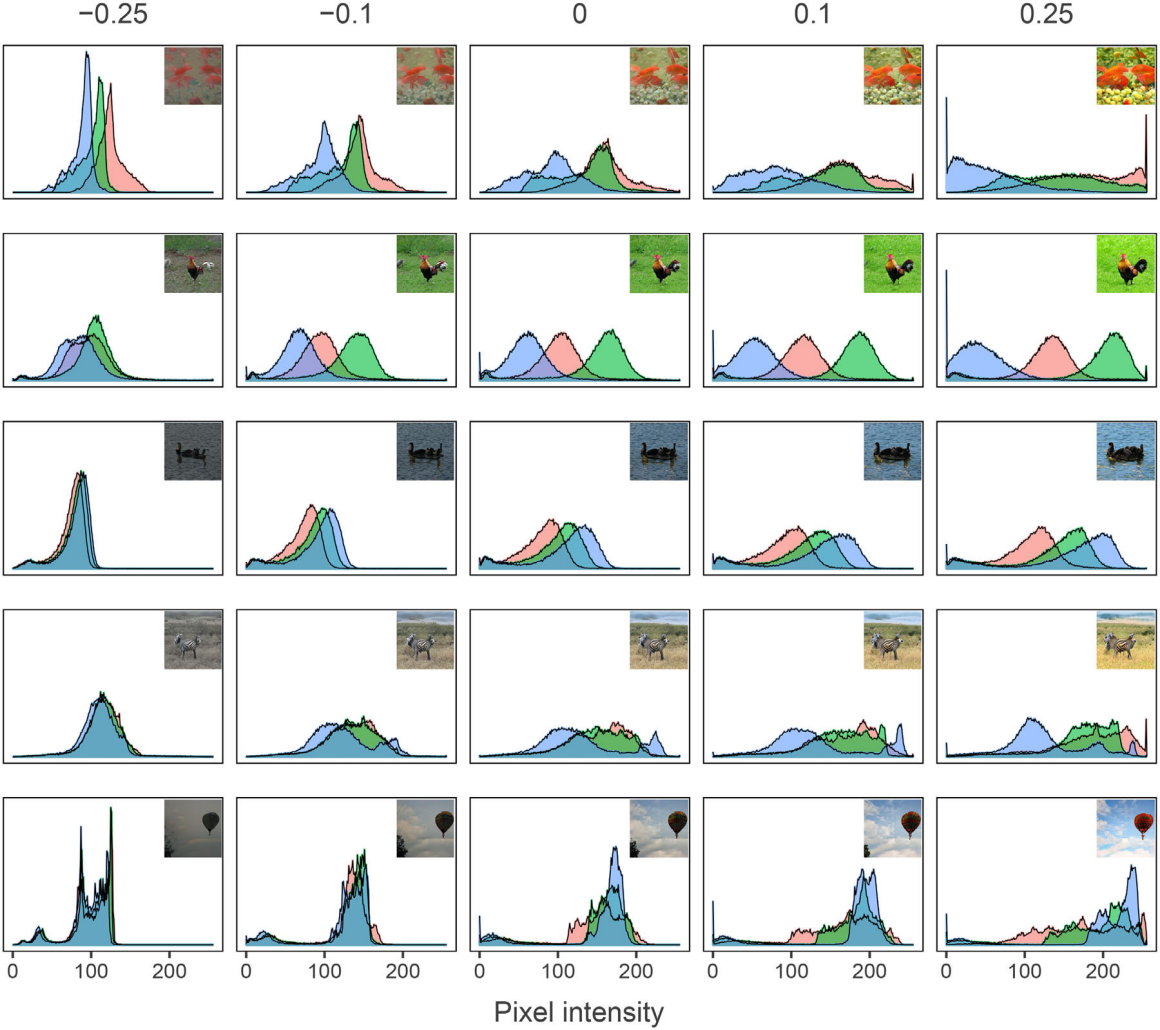
Color distributions. Figure depicting the distributions of RGB pixel intensities for five *α*-values of five image sequences. The image corresponding to the distribution is embedded in the top right of each histogram.

The changes in color distribution for five random image sequences can be seen in Figure 8. For the low *α*-values, the colors tend to be clustered together on the lower end of the scale, indicating mostly dark gray colors. As the *α* increases, the color channels generally become wider and shift more to extreme values, either high or low. This means that the images are getting a larger diversity of colors that are also more intense.

**Figure 9.**
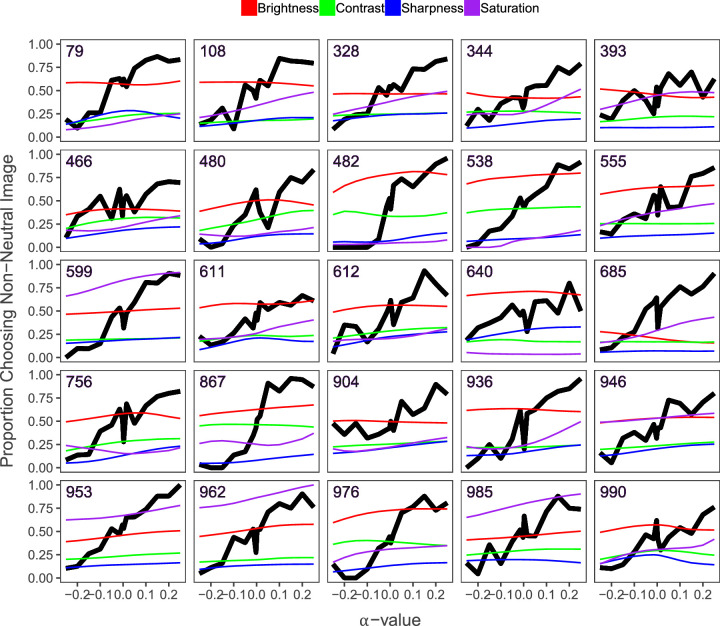
Stable low-level features. Figure depicting the 95th percentile of image sequences with the smallest sum of differences in low-level features from α = −0.25 and α = 0.25. The black line depicts the measured aesthetic quality of each image in the sequence for the different *α*-values. The colored lines represent values for the low-level image features and the numbers in the top left represent the image sequence each graph corresponds to.

Figure 9 shows the image sequences with the smallest changes in low-level features. In all of these image sequences, the aesthetic value of images increases along with *α*, but the low-level features stay relatively stable, indicating that there are changes happening in the images that are not captured by these low-level features.

**Figure 10.**
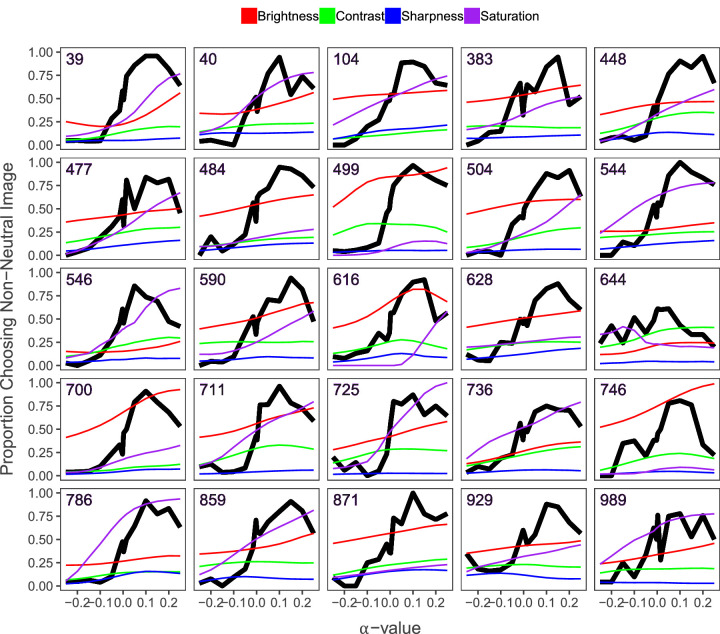
Decreasing aesthetics for high *α*-values. Figure depicting the 95th percentile of image sequences with largest difference in aesthetic evaluation between the highest *α*-values. The black line depicts the measured aesthetic quality of each image in the sequence for the different *α*-values. The colored lines represent values for the low-level image features, and the numbers in the top left represent the image sequence each graph corresponds to.

Figure 10 shows the image sequences with a notable decrease in aesthetic quality of images for the highest *α*-values. While the aesthetic quality of the images decreases near the end, the low-level features all show an increasing trend.

**Figure 11.**
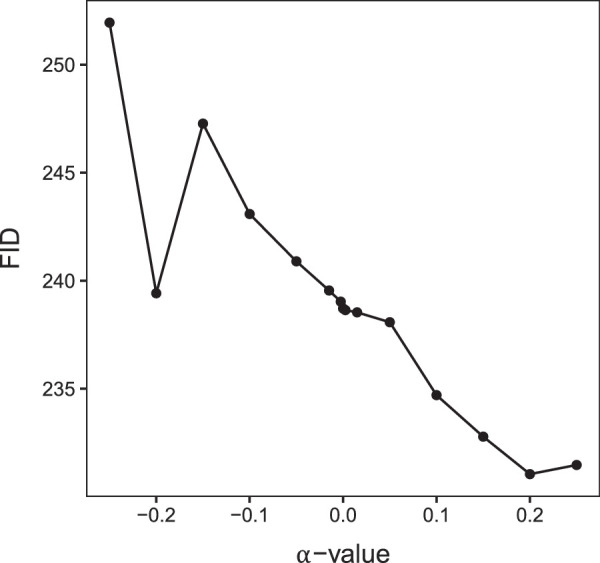
FID scores for *α*-values. Figure depicting the change in measured mean FID scores for each *α*-value. A lower FID represents a higher level of realism.

In Figure 11, the mean computed FID for each *α*-value can be seen. The FID is steadily decreasing the higher the *α*, meaning that the images are getting more realistic as *α* increases.

## Discussion

### Validation

Our behavioral experiment showed that GANalyze was indeed able to generate images of increasing aesthetic value, as seen in the positive slope between the proportion agreement with the neural network and the *α*-values. Even when forced to choose between two nearly identical images, our participants picked the image GANalyze generated to be more beautiful at rates above chance. The relation between GANalyze's *α*-values and our participant's preference followed the shape of a typical psychometric curve in 2AFC discrimination tasks, implying that the internal values of the neural network translate to human aesthetic appreciation, similar to how, for example, auditory stimulus intensity translates to human auditory perception. Furthermore, the low- and mid-level features on their own were able to account for 52% of the observed variance in the aesthetic appreciation of images across categories. Taken together, these findings hint toward the existence of objective properties of beauty, which GANalyze has learned to manipulate. As we have confirmed that GANalyze is indeed able to manipulate these aesthetic qualities in images, we can now use these images to find what makes an image beautiful.

### Demographic effects

Through structural equation modeling, we have determined that while a shortened version of the AEQ is valid, the measured aesthetic experience does not influence the appraisal of image aesthetics in a behavioral experiment (Appendix B). Age and sex also have no effect on neither aesthetic experience nor the aesthetic judgment in the experiment. Nationality, on the other hand, may have a small effect on the aesthetic experience, which was not found in the original study on the AEQ's development by Wanzer et al. (2020). There was no effect of nationality on aesthetic judgment, however. Taken together, these results seem to point toward the objective properties of beauty. However, because our analysis was on the group level, we cannot make any claims on the effects of subjective variables. Furthermore, it is also possible that our aesthetic judgment outcome variable was not specific enough to detect the effects the latent aesthetic experience may have had.

### Behavioral findings

We found that while our participants were more likely to agree with the network for higher *α*-values, their responses to high *α*-values were somewhat less in agreement than those to low *α*-values. To clarify this asymmetry: Images that the network generated to be much less aesthetic than the neutral image were indeed rated much worse: At the most extreme value, namely α = −0.25, our participants agreed with GANalyze 93% of the time that the image with α = 0 (i.e., the neutral image) was more beautiful than the image with α = −0.25. From this we can conclude that GANalyze is very capable of making images *less* aesthetic. When we look at the positive *α*s, we see a similar pattern. However, for the most extreme value, α = 0.25, the agreement was at 81%. While we can still conclude that GANalyze is capable of making images more beautiful, the effects are less pronounced.

It is not immediately clear whether this effect is caused by properties of human perception or GANalyze's parameters. To investigate potential causes stemming from human perception, we will first illustrate a number of relevant theories from cognitive and biological psychology, after which we will situate our finding in the broader theoretical context. Second, we will look into the possibility that it is the generated stimuli themselves that have caused this asymmetry.

#### Perception-based explanation

A supernormal stimulus in evolutionary and biological psychology is an exaggeration of a natural stimulus that evokes a stronger response than the natural one. We encounter these supernormal stimuli everywhere in daily life, from advertisements to propaganda (Barrett, 2010). It follows logically that applying stimuli such as these to art would evoke a stronger response. Costa and Corazza (2006), for example, found that people indeed have an aesthetic preference for exaggerated facial features such as roundness of eyes and lips. Ramachandran and Hirstein (1999) introduced a framework, the peak shift principle, in an attempt to “explain art.” The peak shift principle builds upon supernormal stimuli to claim that art is characterized by caricatures, referring to examples such as prehistoric cave paintings and the Venus “fertility” figures (Ramachandran & Hirstein, 1999). In this framework, amplifying the distinguishing features of an object, even to the point of appearing completely unnatural, should result in higher aesthetic value. This is a controversial framework, however, as it has been claimed to be made up of overly ambitious ideas, flawed argumentation, and unsubstantiated claims (Hyman, 2010). While we have generally found an effect that seems to be in line with that framework in our study, the asymmetry of the positive and negative *α*s raises questions.

From a peak shift perspective, it does not make sense that our participants show a decreased effect for the high-*α* images, as the framework would have predicted that the aesthetic reaction to the (perhaps unnaturally) exaggerated features of α = 0.25 images should be at least as strong as for α = −0.25 images. The asymmetry we found then implies that there is likely more to it than Ramachandran and Hirstein (1999) had proposed. We initially hypothesized that perceived realism might be an explanation for this pattern as extremely high *α-*images would be perceived as artificial-looking. However, after computing FID scores, this does not seem to be the case.

Approaching our findings from the perspective of the processing fluency theory described in the Introduction (Reber et al., 2004), this would imply that the images generated with the highest *α*-values are processed at similar rates, regardless of the changing low-level features. Intuitively, it makes sense that the low-level features initially boost processing fluency. At a certain point, however, these objective changes may be overshadowed by subjective factors playing a relatively bigger role. For example, some of the high values of contrast or saturation may give the images a highly unnatural or artificial look, pushing them beyond the usual range of image values. They may no longer fit the expected values for a particular type of image. These subjective factors are beyond the scope of this study, however, and require further research to be understood more fully.

#### GANalyze-based explanation

It is also possible that the asymmetry was caused by the stimuli themselves rather than human perception. Inspecting the trajectories of the contrast and sharpness in Figure 6 shows that their effects slow down for the high positive *α*-values. This could be the reason for these particular images not being rated much higher than those of adjacent lower *α*-values. Why exactly GANalyze reduced the effects of contrast, sharpness, symmetry, and visual complexity is unclear. Perhaps the GAN had actually learned that unrealistic images with extreme image features are not appraised positively and therefore reduced the effects of these features. But this hypothetical compensation seems not to have been very effective, as the extreme high *α*-images are still rated lower than expected. In addition, when looking at the effects of image features on aesthetic appraisal across categories, we see that the aforementioned features still increase along with aesthetic appraisal.

Another possible explanation comes from the influence of higher-level image features. Examples of such features are “harmony” and “balance” but also Gestalt principles such as figure-ground segmentation or the relative convexity and sharpness of the central object compared to the background. While these factors are not directly represented in any of the low-level features themselves, they may be disturbed by extremely high contrast and sharpness, or symmetry and visual complexity. It is possible that GANalyze has learned to prioritize these resulting higher-level features over the lower-level features separately. The increase in lower-level features we observe in our results is then a consequence of GANalyze improving these higher-level features.

### A visual definition of beauty

**Figure 12.**
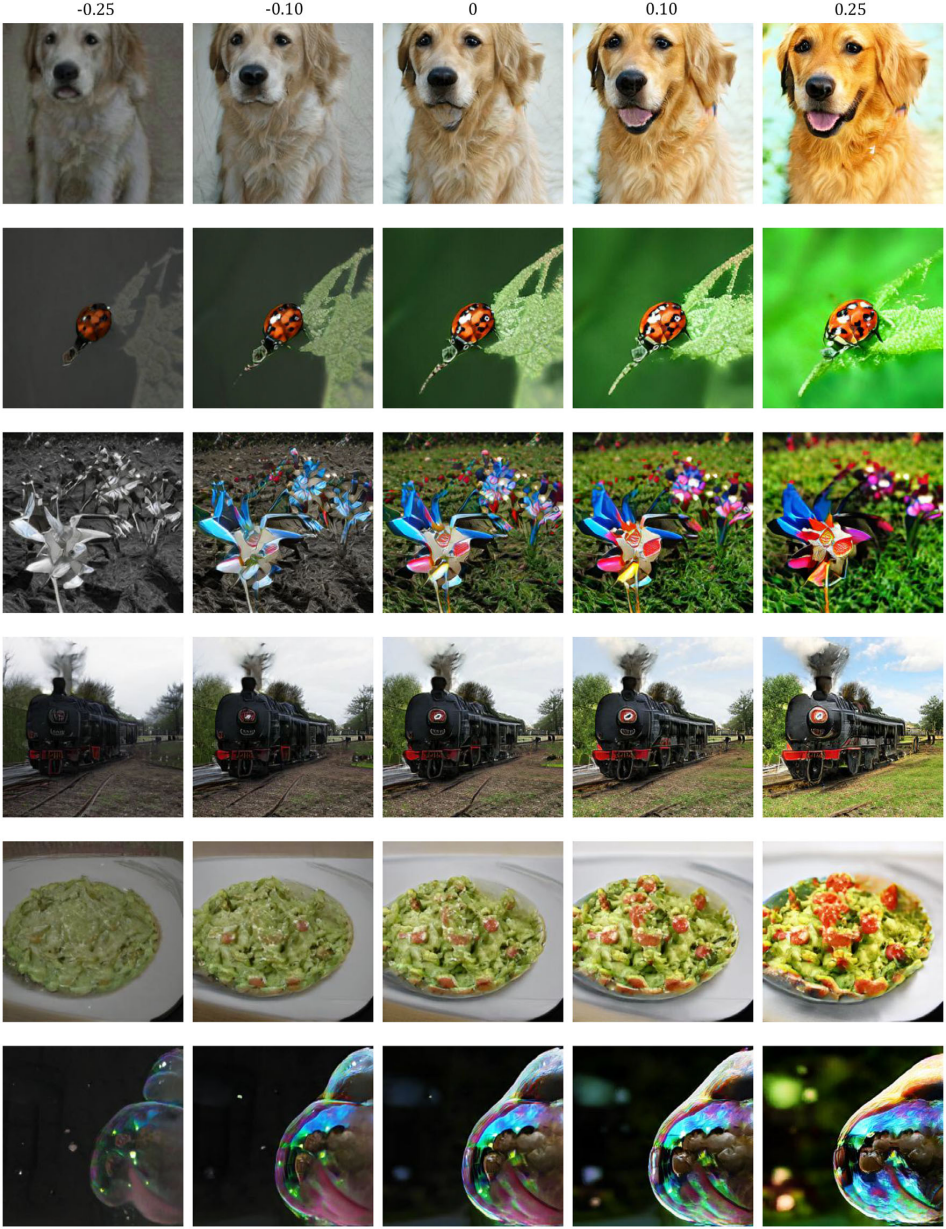
Visual definition of beauty. Our visual definition of beauty presented as the increasing aesthetic value of an image. Six image sequences are shown with five *α*-values each.

Just as Goetschalckx et al. (2019) used GANalyze to make a visual definition of memorability, we made a visual definition of beauty. Our visual definition can be seen in Figure 12. We quantified the changes made to the images in four image features as suggested by Ke et al. (2006) and three additional mid-level features shown in Figure 6. All of the image features we studied increased along with the *α*-values. It would be naive, however, to claim that simply increasing the brightness, contrast, sharpness, and saturation of any given image would increase its aesthetic quality.

We have reasons to believe that instead of simply increasing these parameters, GANalyze instead used sophisticated methods acquired through deep learning to determine which features to modify, how intense the modification should be given a certain *α*-value, and how the different image features interact with each other. For example, looking at the color distributions for five image sequences in Figure 8 shows that while there is indeed a pattern of moving toward the extremes of the spectrum, the way this happens differs between the image sequences. The most prominent colors tend to increase while the others decrease, but this differs for every image.

#### Low-level image features

##### Brightness

The mean pixel values of grayscale images increased along with the *α*-values, meaning that the images got brighter. While this is consistent with the literature, brightness in the context of aesthetics is often evaluated as relative brightness of the main object compared to the background (Ke et al., 2006; Obrador, Schmidt-Hackenberg, & Oliver, 2010), which we have not done in this study. A visual inspection of the image sequences, however, does not seem to indicate specific changes to the relative brightness, as everything seems to increase together. It is unclear if relative brightness is not as important as the literature makes it out to be, or GANalyze simply failed to implement it properly.

##### Contrast

The spread of the grayscale pixels values grew, meaning that the contrast of the images increased. Contrast has also generally been found to correlate with aesthetic appraisal (Wong & Low, 2009), although researchers usually tend to differentiate between lightning contrast and color contrast, which we have not considered in the present study.

##### Sharpness

The sharpness of images increased, which is also a common finding (Redi, Rasiwasia, Aggarwal, & Jaimes, 2015). Interestingly, for some images, GANalyze made the main subject sharper and added blur to the background, which is a more advanced transformation than simply making everything sharper. Sharpness in this sense contributes to the main subject of the image being featured more prominently, which is an important factor in aesthetic appraisal (Wong & Low, 2009).

##### Saturation

Studies generally find that increasing the saturation of the main subject of an image is important for its aesthetics (Wong & Low, 2009), although from a visual inspection, GANalyze seems to increase the saturation for the image as a whole. However, because we did not measure the saturation of the main subject formally, we cannot confidently claim that GANalyze ignores this property.

#### Mid-level image features

**Figure 13.**
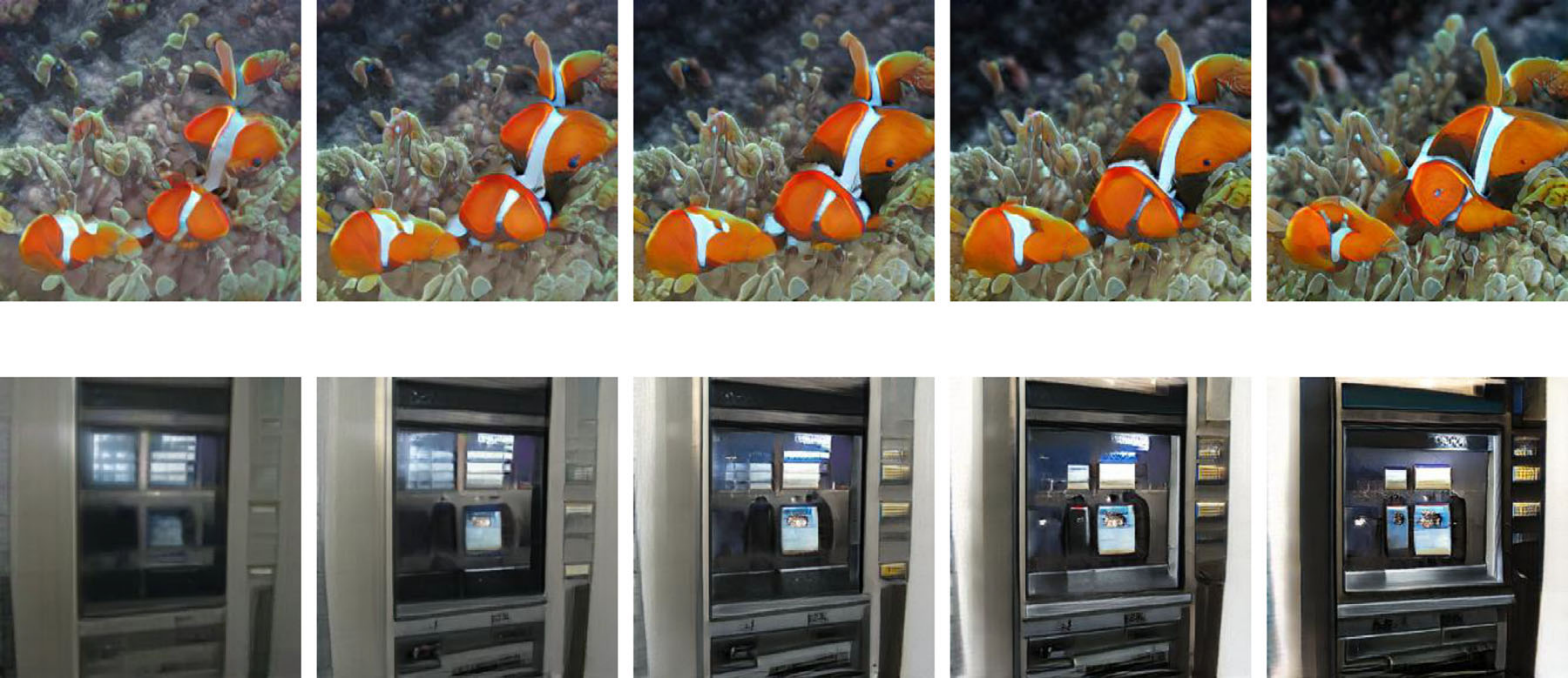
Image sequences 393 and 480. Image sequences depicting goldfish (top) and an ATM (bottom). Presented from left to right are α = −0.25, α = −0.10, α = 0, α = 0.10, α = 0.25.

We investigated colorfulness, visual complexity, and symmetry as mid-level features. We found that just as with the low-level features, they all increased along with the *α*-values. However, when we tested the effect of all features in a linear model, we found that only symmetry was not significant. This may be explained by the fact that the correlation between measured visual complexity and symmetry was 0.81. We believe this is caused by the fact that both measures are computed using a form of quadtree decomposition. In addition to these computed features, we expand our analysis of mid-level features by adding some observations and interpretations based on the patterns found in Figure 9 and Figure 10. Because the aesthetic value of the images increases while the low-level features stay relatively stable in Figure 9, there must be another factor at play. For example, inspecting image sequences in Figure 13 shows that the low-level image features do not change much as *α* increases. However, our participants much preferred higher over lower *α*-values nonetheless. While we cannot give a clear quantitative explanation with the present study, it seems that—in addition to minor changes in low-level features—the overall composition may become better organized.

**Figure 14.**
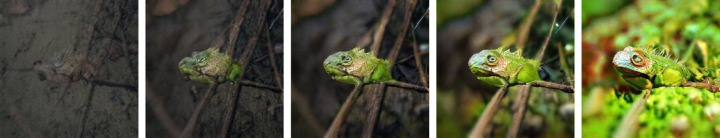
Image sequence 39. Image sequence depicting an iguana. Presented from left to right are α = −0.25, α = −0.10, α = 0, α = 0.10, α = 0.25.

In Figure 10, we can see that the aesthetic value of images decreases for the highest *α*-values while the low-level image features steadily increase. This corroborates our hypothesis that increasing the low-level features too much may cause disturbance in some higher-level organizations. However, as our images tend to become more realistic as *α* increases (Figure 11), we may assume that extreme low-level features are not affecting the realism much. An example of how superfluous low-level features may lower the aesthetic value can be seen in Figure 14. While the final image has the highest values for the low-level features, our participants did not find it the most beautiful. The last image may have poorer figure-ground organization as the color of the background has also been increased a lot, suppressing how well the animal stands out from the background in the one-before-last image and reducing the visibility of the parallel elongated twigs, which contributes to the attraction of the one-before-last image.

#### Visual inspection

Simply looking at the image sequences from our visual definition in Figure 12 can give us some insight into the higher-level image features that change together with the *α*-value. The first row, for example, shows the changes in low-level image features we previously discussed. However, a notable change from α = −0.25 to α = 0.25 is the fact that the dog's emotional expression seems to change from sad or neutral to happy with its tongue out. This is an example of a change that is impossible to quantify with only the low-level features we analyzed. It also demonstrates the potential capabilities GANs have for studying high-level cognitive properties. The fourth image sequence depicting a train shows a less pronounced effect. While the low-level image features do increase, the changes are not as obvious as in the other sequences. The sixth image sequence, depicting a bowl of presumably guacamole, also shows an interesting effect on top of the low-level image features. As the *α* increases, more ingredients seem to appear.

While the patterns found in these visual inspections can be subjective, they do show that GANalyze and, as a consequence, the aesthetic value of images rely on more than just low-level image features. In other words, to make an image more beautiful, one has to do more than simply make them sharper and more colorful. There is an argument to be made that colorfulness is the most salient change, and the results of our statistical models agree. However, these models also show that the other features still significantly contribute to the aesthetic appreciation of an image. Colorfulness by itself explains 32% of variance in aesthetic appreciation and 41% in *α*-value. The fact that adding the other predictors significantly increases these values (to 52% and 62%, respectively) shows that they must also add changes that contribute to the aesthetics of an image. To illustrate this, the two example image sequences in Figure 13 do not show much change in colorfulness while they still increase aesthetic appreciation. In general, the changes made to the higher-level features differ between each image, making it difficult to highlight the factors in question separately with our present resources.

### Limitations

One limitation is that our experiment's design primarily relied on an objectivist conception of beauty. We did not pay much attention to individual differences in aesthetic appraisal other than broadly comparing the effects of demographic variables on the agreement with GANalyze. As it is believed that perception of beauty is an interaction between subjective and objective variables, it may prove beneficial to conduct a deeper investigation into the subjective part of the equation. For example, it may be possible to group images based on the most saliently changing low-level feature. The steepness of the psychometric functions may then be compared between individuals for each of the image features.

Another limitation of our work is a bias in these photography data sets that the components of GANalyze were trained on. The generator of GANalyze was trained on ImageNet, and the assessor was trained on AADB. Although both data sets consist of amateur and professional photographs, one could argue that there is always a bias toward more aesthetic images since we tend to only share our most aesthetic photos on the Internet. This is a bias that any study in computational aesthetics is subject to, however.

## Conclusions

The goal of this study was to find out what makes an image beautiful and whether we can use a GAN as a means to answer this question. To do so, we trained GANalyze to generate images of increasing aesthetic value. Using these images, we validated the GAN's understanding of aesthetics, allowing us to utilize these images to find features that underlie the aesthetic value of an image. With these images, we constructed a so-called visual definition of beauty. By extracting low- and mid-level features from these images, we found that brightness, contrast, sharpness, saturation, colorfulness, and visual complexity were properties that consistently made images more beautiful.

In a broader sense, we showed that GANs have a potential future in studies on aesthetic appreciation. A significant benefit of GANs is that they can be used to generate rich stimuli that are easy to manipulate and seem to be, as we have shown in this study, ecologically valid. In a philosophical sense, we provided additional evidence for objective properties of beauty.

In addition, our study did not delve quite deep enough into the subjective features of aesthetic appraisal.

GANs and artificial neural networks in general are proving to be very useful in conducting research on aesthetics and psychology as a whole. These powerful new methods may prove to be a crucial tool leading to groundbreaking discoveries in cognitive psychology (Goetschalckx et al., 2021).

## Figures and Tables

**Figure 15.**
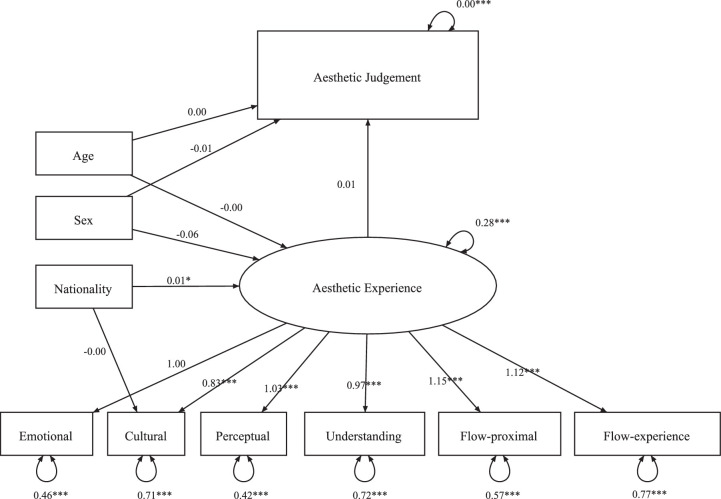
Final MIMIC model. Rectangles are depicting observed variables while the oval is depicting the latent variable of interest. The numbers attached to each arrow represent the factor loadings. For the structural model, factors have been standardized on “Emotional.” *p < 0.05, **p < 0.01, ***p < 0.001.

## Appendix A

Shortened Aesthetic Experience Questionnaire

**In general, when I view art** **. . .**

1. I experience a wide range of emotions. (emotional)
2. I compare the past culture of the art with present-day culture. (cultural)
3. The composition of a work of art is important to me. (perceptual)
4. I try to understand the work completely. (understanding)
5. I have a clear idea of what to look for when viewing the work of art. (flow-proximal)
6. I lose track of time when I view the work of art. (flow-experience)

## Appendix B

### Structural equation modeling

Our structural model needed to have a latent variable measuring aesthetic experience, which also had to be properly explained by the observed variables. In addition, we wanted to know whether age, sex, and nationality affected this. Furthermore, we were interested in the effects of aesthetic experience, age, sex, and nationality on the outcome variable of the behavioral task measured by the proportion of agreement with the neural network. To test this, we used structural equation modeling (SEM) with the lavaan package (Rosseel, 2012) in R version 4.1.0 (R Core Team, 2021) to build and evaluate models based on theory that would give an explanation to the data. All models were fitted using a maximum likelihood estimation method, and the fits were evaluated with the comparative fit index (CFI), Tucker–Lewis index (TLI), and the root mean square error of approximation (RMSEA).

First of all, we performed a priori confirmatory factor analysis (CFA) to validate whether our shortened version of the AEQ was indeed sufficient in explaining the latent aesthetic experience variable. We used the “emotional,” “cultural,” “perceptual,” “understanding,” “flow-proximal,” and “flow-experience” scales as indicators for our latent variable.

Next, we moved on to more complicated structural models to better describe the full data set. More specifically, we created a multiple indicators, multiple causes (MIMIC) model aimed to show whether age, sex, and nationality affected the latent aesthetic experience variable. Crucially, we were interested in the effects of the aesthetic experience latent variable and age, sex, and nationality on the outcome of the image comparison behavioral task. We used the proportion of agreement with the GAN as the measure for aesthetic judgment here. Our initial idea for this model consisted of the latent variable, explained by the indicators, affecting aesthetic judgment in the behavioral task. Age, sex, and nationality would have an effect on both the latent variable and the behavioral outcome of judgment. We also made another alternative model based on different theoretical arguments. For this second model, we decided that it did not make sense for nationality to have a direct effect on aesthetic judgment, so we removed that effect and instead added an effect of nationality on the cultural scale from the AEQ, as we believed this would make sense. These models would additionally test the questions Wanzer et al. (2020) had regarding their homogeneous population sample.

Even though the new MIMIC model did not appear to be significantly better or worse than the older one, we decided to keep the newer model as it made more sense from a theoretical point of view.

### Results

Our first model, the CFA on the shortened AEQ, resulted in a validation of the shortened questionnaire. The model fit can be seen in Table 3 under CFA. All fit indices, apart from the conservative χ^2^, were in the acceptable range (i.e., >.90 for CFI and TLI, and <.08 for RMSEA). All standardized factor loadings from the latent variable on the measured indices were significant (*p* < 0.001) and strong enough to justify interpretation (>.80). We interpret these results as evidence for our shortened version of the AEQ being adequate for measuring an individual's aesthetic experience.

**Table 3.** Model fit indices. *Notes*: ****p* < 0.001.

Model	χ^2^	*df*	CFI	TLI	RMSEA
CFA	33.92***	9	.96	.93	.07
MIMIC 1	68.72***	29	.94	.91	.05
MIMIC 2	68.00***	29	.94	.91	.05

For our first MIMIC model, we also obtained good fit measures, as seen in Table 3 under MIMIC 1. This model included an effect of sex, age, and nationality on aesthetic experience and an effect of sex, age, and nationality on the proportion of agreement with the GAN. While the measurement part of the model remained largely unchanged, the new structural part of the model only showed a significant effect of nationality on aesthetic experience (β = 0.01,  *p* < 0.05). This finding may have consequences for the AEQ itself, as Wanzer et al. (2020) had mentioned that their own sample only included U.S.-based participants. On the other hand, we did not find an effect of age and sex on aesthetic experience, unlike Wanzer et al. (2020). The most important finding here is that there was no effect of the latent aesthetic experience on the behavioral outcome variable of aesthetic judgment.

For our second MIMIC model, illustrated in Figure 15, we made changes on theoretical bases. The fit indices were equally good, as seen in Table 3 under MIMIC 2, but we believe this second model makes more sense from a theoretical point of view. The standardized factor loadings were again very similar. Again, the effects of age and sex on aesthetic experience and aesthetic judgment were not significant. Nationality still has a significant effect on aesthetic experience here, but not on the cultural indicator. There was also no significant effect of aesthetic experience on aesthetic judgment here.

We can interpret these results as there being no effect of a person's demographic characteristics and their aesthetic experience on the outcome of the behavioral image rating task. This implies that the simple image comparison we used in our experiment is not influenced by seemingly relevant factors, pointing to a potential universal nature of aesthetic appreciation of the images we provided in the task. Furthermore, this will make our interpretations of the factors determining the aesthetic value of an image through GANalyze more generalizable.

## Appendix C

### Task Instructions

For this study, a generative adversarial network (GAN) was trained to make images more beautiful. The goal of this study is to test whether this network really has some “understanding” of aesthetics.

Before the task, you will be asked to indicate how much experience you have with art. In addition, demographic information linked to your Prolific account (such as age, sex, nationality, etc.) will be linked to your responses.

During the task, you will be presented with two similar-looking images produced by the GAN. The images are assumed to be of differing aesthetic quality (according to the GAN). Your task is to indicate which of the two images you believe is more aesthetic. You indicate this with the key on your keyboard corresponding to the location of your preferred image. If you prefer the left image, you press F on your keyboard, and if you prefer the right image, you press J.

Because this study aims to test the GANs’ understanding of aesthetics, it is important that you pick the image that you really feel is better looking and not what you expect the GAN to produce. The task will be 10 minutes with a short break after 5 minutes. You should not take too much time during each trial, and it is recommended to pick whichever image you prefer at first glance without analyzing too many details.

If you press at random during the whole experiment or provide irrational data in another way, your submission will be rejected and you will not get paid.

This study has been approved by the Social and Societal Ethics Committee of the KU Leuven (SMEC).

In case of complaints or other concerns with regard to the ethical aspects of this research, you can contactsmec@kuleuven.be.

If you continue to the experiment, you will have given consent for participation and data collection.
